# The diagnosis and treatment of primary vitreoretinal lymphoma: a review

**DOI:** 10.1186/s40942-018-0120-4

**Published:** 2018-05-07

**Authors:** Jose S. Pulido, Patrick B. Johnston, Grzegorz S. Nowakowski, Alessia Castellino, Harish Raja

**Affiliations:** 1 Department of Ophthalmology, Mayo Clinic, 200 First Street, SW, Rochester, MN 55905 USA; 2 Departments of Hematology, Mayo Clinic, Rochester, MN USA; 3 Department of Molecular Medicine, Mayo Clinic, 200 First Street, SW, Rochester, MN 55905 USA

**Keywords:** Vitreoretinal lymphoma, Diffuse B cell lymphoma, MYD88 L265P, Ibrutinib

## Abstract

**Background:**

To describe the recent diagnostic and treatment options for the most predominant form of primary vitreoretinal lymphoma (PVRL), namely diffuse large B cell lymphoma. This is mainly based on the experience at the Mayo Clinic as well as a partial review of the literature. MYD88 L265P mutation is seen in about 80% of cases; therefore, a polymerase chain reaction for this mutation helps in making the diagnosis that has been notoriously difficult to make. Local therapy using intravitreal methotrexate and rituximab has been very helpful in the treatment of the local disease. Systemic high-dose intravenous methotrexate is helpful in treating bilateral disease in conjunction with intravitreal therapy. Whether it is helpful in preventing or delaying the development of central nervous system lymphoma (CNSL) is still in dispute. If there is development of CNSL or recurrent ocular disease, alternatives to high-dose methotrexate under investigation include pomalidomide, stem cell transplantation, or ibrutinib, with or without local therapy. Vitrectomy alone might be helpful as a debulking procedure. Because of the risks of redevelopment of disease, local radiation should be given if other options are not possible. Aqueous levels of IL10 are helpful in following the redevelopment of local disease.

**Conclusion:**

Although PVRL is still a difficult disease to diagnose and treat, new advances are helping to make these easier. Larger collaborative studies will be helpful in determining better treatments.

## Background

Primary vitreoretinal lymphoma (PVRL) is a great masquerader and, many times, has been first considered to be uveitis before the final correct diagnosis was made. In the 1980s and 1990s, PVRL was called reticulum cell sarcoma. It was thought to be very malignant, and mortality was very high with median survival of less than 1 year [1, 2].

Unfortunately in the literature, PVRL has sometimes not been well-distinguished from other forms of intraocular lymphomatous involvement, causing confusion between the treatments amongst the different forms of intraocular lymphoma.

Intraocular lymphomas can be separated into vitreoretinal lymphomas, choroidal lymphomas, and iridial lymphomas [3]. Vitreoretinal lymphomas (VRL) may then be subdivided into primary or secondary. Secondary VRL are associated with systemic lymphomas elsewhere either in the body, especially in the testes since it also is an immune privileged site or the brain [4]. Only a minority of cases of PVRL are T cell lymphomas, and the other 95% are diffuse large B cell lymphomas (DLBCL) [3, 5–7]. The cells in DLBCL are CD20 +, which is helpful in diagnosis as well as in consideration of treatment.

Choroidal lymphomas differ from vitreoretinal lymphomas. As opposed to VRL cells, which are present in the vitreous, the subretinal space anterior to Bruch’s membrane and in the retina, choroidal lymphomas do not penetrate through Bruch’s membrane and, thus, are present posterior to Bruch’s membrane [8].

This is important since VRLs are protected by the blood-retinal barrier while choroidal lymphomas are not. Additionally, primary choroidal lymphomas tend to be low-grade B cell lymphomas, typically marginal zone B-cell lymphomas, though some rare cases are choroidal DLBCL [9]. Iridial lymphomas tend to be diffuse large B cell lymphomas [10].

### Diagnosis

Through the years, the diagnosis of PVRL has been very difficult and, thus, treatment has been delayed. The delay has caused some patients to develop central nervous system (CNS) involvement, resulting in the need for systemic treatment [11, 12]. On average, it takes 3 months between the initial CNS symptoms and the diagnosis of CNS lymphoma [11]. In contrast, for PVRL, on average, it takes over 1 year between symptom onset and the diagnosis of PVRL [11, 13].

In this time period, it is common for the patient to have had multiple eye examinations. They have been considered to have uveitis (ocular inflammation) and, many times, have been treated with corticosteroids. Since corticosteroids are lympholytic, there is a transient beneficial effect; therefore, the ultimate diagnosis is further delayed. In addition, if the corticosteroids are given before a vitreous biopsy, fragile diffuse large B cells are lysed, further delaying the ultimate diagnosis.

**Fig. 1:**
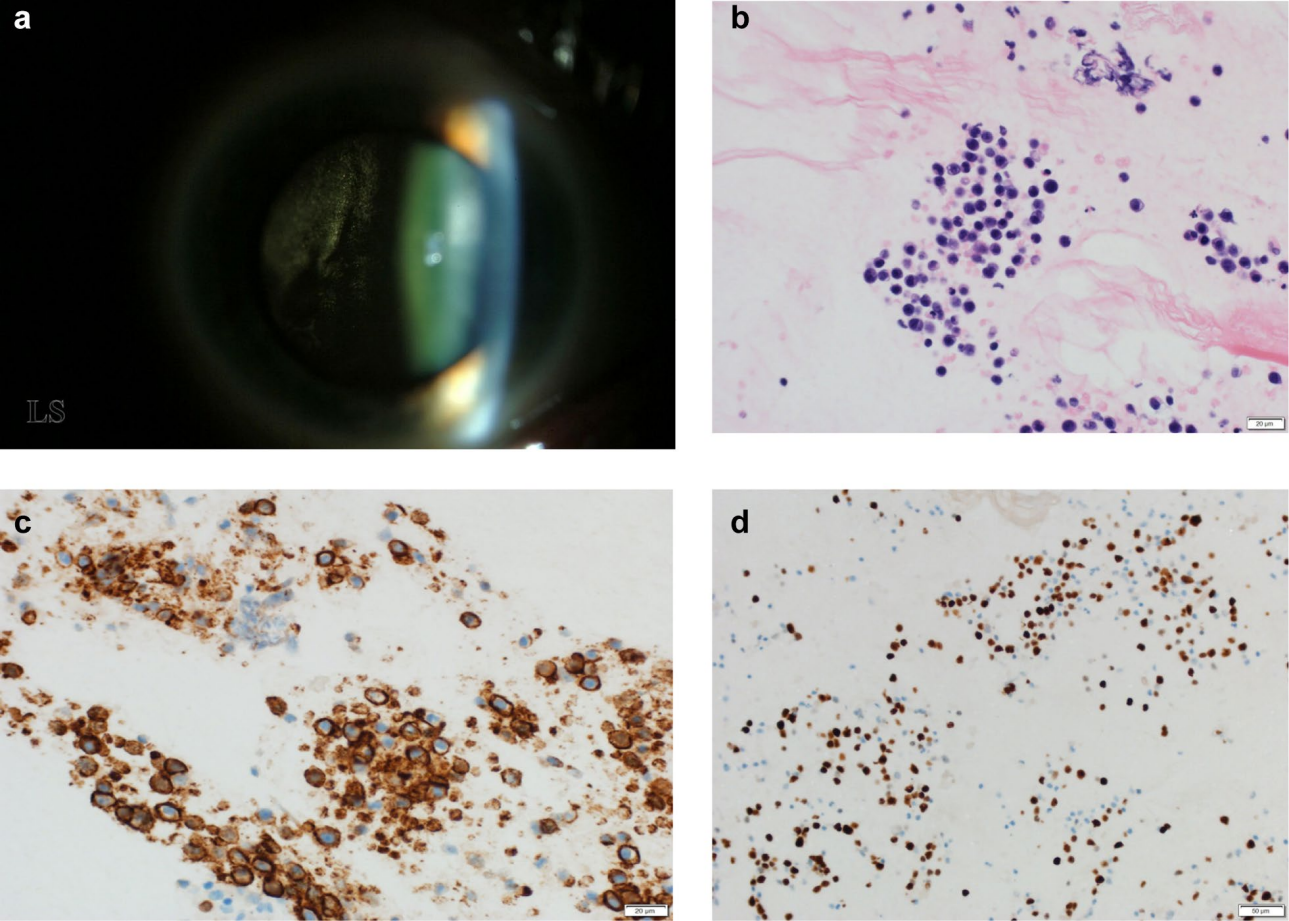
Diffuse large B cell lymphoma in the vitreous of a patient (**a**). Cytomorphology of the vitreoretinal diffuse large B cell lymphoma cells (H&E ×400—photograph thanks to Diva Salomao, MD (**b**). Immunostaining for CD20 of the vitreoretinal lymphoma cells (×400) (**c**). Ki-67 staining of the vitreoretinal cells; note the extensive staining which is consistent of marked local replication (×200) (**d**)

To make a diagnosis, it is important to determine the region of the eye with the most cellular involvement with lymphoma. The anterior chamber rarely has many cells; therefore, sampling of the anterior chamber is not usually advised. The eye that has the most vitreous cells should be biopsied; and if the patient has been placed on systemic or local corticosteroids, it is best to wait a few weeks to allow more of a vitreal infiltration before doing a vitreous biopsy. In the meantime, if an MRI (magnetic resonance imaging) of the brain has not been done in the last 2–3 months, it should be performed a few weeks after stopping the corticosteroids to make sure no CNS lesion has developed (Fig. 1). With small-gauge vitrectomy instruments, the risks of the vitreous biopsy have been decreased significantly. It is still important to close the sclerotomies with an absorbable suture to avoid the possibility of cells leaking out. In addition, valved trocars should be used for the same reason. Obtaining a good vitreous biopsy is critical. Two methods can be utilized. In both, a cut rate of about 600 cuts per minute with a 25-gauge vitrector is used. A 12-cc syringe and manual aspiration can be used, and a wide-field viewing method is very helpful. In one method, air is infused while the vitreous is removed. About 2 cc of pure vitreous can be obtained with this method. In the other, saline infusion is turned on and off, while the vitrector is moved from vitrectomized area to non-vitrectomized area for a 6-cc partially diluted sample to be obtained. It is critical that the pathologist and their laboratory are aware ahead of time that a vitreous sample is being sent for examination for possible PVRL. In addition, having a pathologist who has experience in ocular cytology and histopathology is essential. Many facilities examine cellular pellets with immunohistochemistry to make a diagnosis. It might be that vitrectomy can have an effect in decreasing tumor burden and thus might have a therapeutic effect [14, 15].

Elevated serum interleukin 10 (IL10) levels are seen in patients with DLBCL and has been shown to be correlated with shorter event-free survival [16]. The effect of IL10 in the retina is to decrease inflammation and, thus, decreasing leakage through the blood retinal barrier similar to its effect in the nervous system [17, 18].

Teleologically, it makes sense that the lymphoma cells would secrete factors that would protect the cells from the immune system. As it is, the cells have honed to the vitreous because it is protected from the immune system. There have been attempts at making the diagnosis by evaluating the interleukin levels in the vitreous or aqueous humor [19].

Interleukin 10 levels or IL 10/IL6 ratios have been used. Interleukin 10 levels tend to be elevated in both the vitreous and aqueous in eyes with vitreoretinal lymphoma [20, 21].

Because of variability in the initial IL10 level in the aqueous, it cannot be utilized for making the diagnosis of VRL. There are no definite cut-offs, so the area under the curve of these tests on a ROC (receiver operator characteristic) curve are low [22]. In addition, there is variability in the ELISA (enzyme-linked immunosorbent assay) levels obtained not only from kits from different companies or laboratories but also between one lot and another from the same company. There are variabilities of normal levels, so only a difference of levels using the same lot number by the same company can be used; this is, therefore, only helpful in following treatment results as discussed below [21, 22]. Additionally, there is significant variability between lots of ELISAs, so care must be taken in interpreting the results.

Some investigators have utilized FACS (fluorescence-activated cell sorting) analysis to look for CD20 + cells [23]. Flow cytometry can identify clonality by “gating” for kappa and lambda light chain positivity and dominance. This is often combined with minimal cytomorphology to make a diagnosis of PVRL. However, since there are not many cells in most cases, the use of FACS can quickly exhaust the sample and may not afford a diagnosis. In addition, the B cells in the vitreous tend to be larger and more fragile than in the blood so that analysis of the morphology and the FACS results can be difficult and non-definitive [24].

Regardless of which of the two methods that the vitreous is obtained, the sample is then diluted with RPMI (Roswell Park Memorial Institute) solution in a 2/1 dilution, and transported immediately on ice to make a cytospin and then a cytoblock. Histology can be performed on the cytoblock to observe the morphology of the cells. Immunostaining of the cells for CD20 and CD3 cells should be performed. Finally, a sample should be sent for an allele-specific PCR (polymerase chain reaction) for the MYD88 L265P mutation that is seen in 60 to 80% of cases of vitreoretinal lymphoma [25–28]. This test can be ordered from the Mayo Clinic labs or any other CLIA-approved laboratory that offers this test. The clinician should call ahead of doing the biopsy to make sure that the laboratory will take the sample and to find out how the sample should be handled.

If the MYD88 allele-specific PCR is positive in the context of CD20 + cells in the vitreous, a diagnosis of vitreoretinal lymphoma can be confirmed. PCR for monoclonal B cell rearrangement can also be done, but more cells are required and IGH rearrangement can have a high false-negative rate; in addition, it requires many more cells than MYD88 L265P mutation testing. Finally, it is much more subjective than the MYD88 testing [25, 29].

**Fig. 2:**
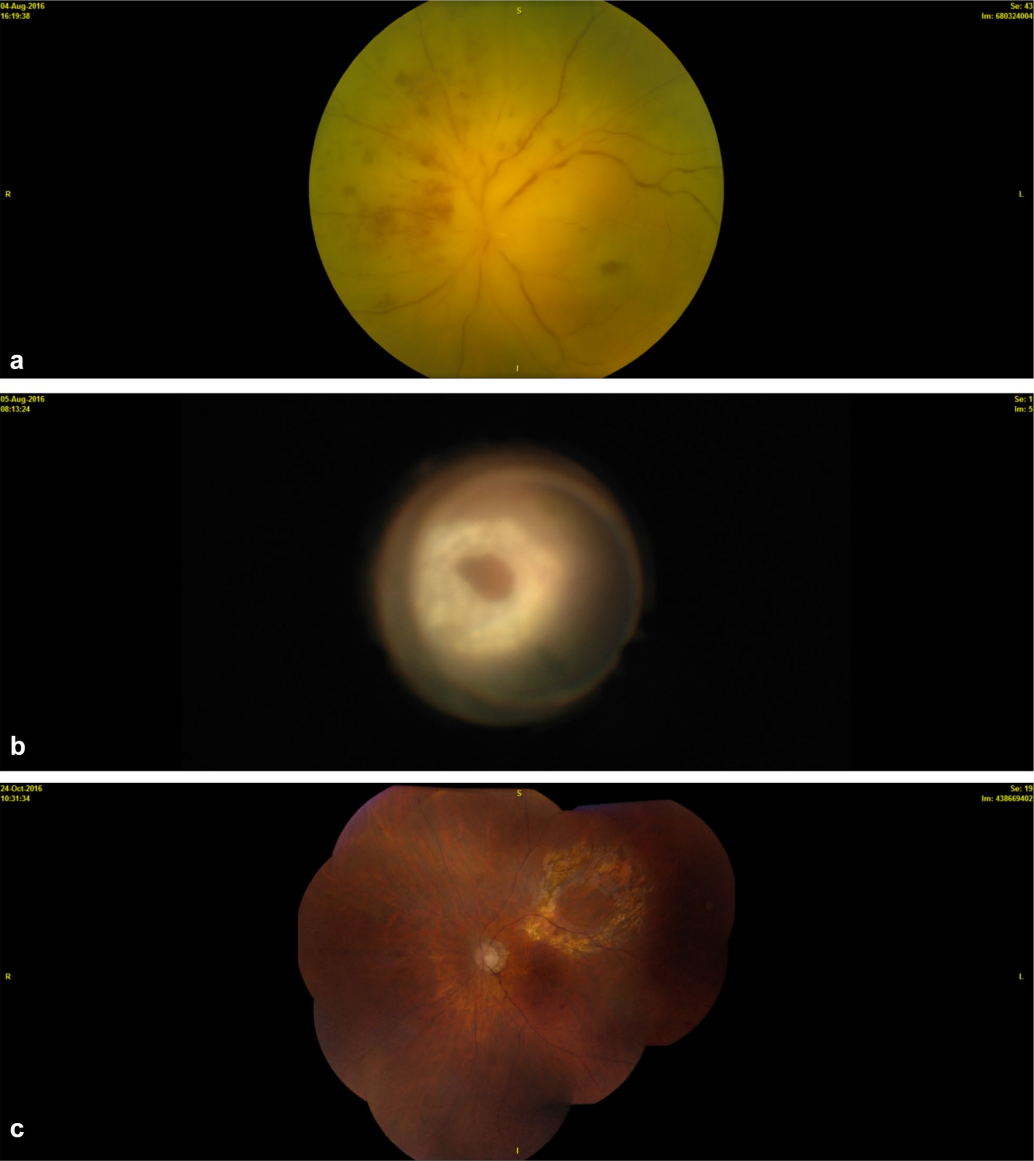
Creamy white retinal involvement by vitreoretinal lymphoma was suspected (**a**) and a vitrector was used to make the diagnosis (**b**). Final photograph following biopsy and treatment with intravitreal methotrexate and rituximab therapy and systemic therapy with ibrutinib (**c**)

If the biopsy is nondiagnostic and there is still strong suspicion of vitreoretinal lymphoma, then the other vitreous should be biopsied; or if there are aggregates of subretinal cells, a retinal biopsy using the same vitrector technique of a lower cut rate and manual aspiration should be done (Fig. 2). Choroidal biopsy is not required since the cells all reside anterior to Bruch’s membrane. Endolaser around the biopsy site can be done as well; however, it should be at the discretion of the surgeon.

Targeted next-generation sequencing (NGS) on small volumes of vitrectomy fluid has promise in aiding the diagnosis of PVRL. A recent publication examined small fluid volumes from four patients with suspected or known VRL and performed NGS spanning 126 genes [30]. Gain-of-function mutations in the MYD88 oncogene were detected including the L265P encoding mutation, as well as a mutation encoding an S243 N mutation. Also identified were losses in the tumor suppressor CDKN2A gene in all four cases and a single copy loss in one sample encoding the tumor suppressor PTEN. Addition of this technology will likely enable more rapid diagnosis of PVRL.

## Treatment

### PCNSL

Considering the similarity between PVRL and PCNSL as well as how much more prevalent is PCNSL, it is worth reviewing the treatments of PCNSL. The possibilities include whole-brain radiotherapy (WBRT), chemotherapy, WBRT and chemotherapy, or high-dose chemotherapy with stem cell therapy (HDT with SCT). WBRT was used in RTOG (Radiation Treatment Oncology Group) 8315 with 36-40 Gy to the whole brain plus a 20-Gy boost to the tumor as well as 2-cm margin around the tumor [31]. It was a phase II trial with 41 patients. The overall rate of remission (ORR) was 90%, with a complete remission of 55% and partial remission of 35%. The median was 12.2 months, and 61% had brain relapse. High-dose methotrexate at 8 mg/m(e)2 (meters squared) has a 55.4-month overall survival rate [32, 33]. For HDT with HSCT (high-dose therapy with hematopoietic stem cell transplantation), the first trial had mildly impressive results [34]. This study used MTX at 3.5 mg/m(e)e)2 plus cytosine arabinoside (ARA C) and Cytoxan followed by BEAM [(BiCNU) carmustine, etoposide, ARA-C—cytarabine, melphalan)] conditioning and allograft stem cell. There were 28 patients enrolled but only 14 proceeding to transplant. The median follow-up was 9.3 months. The compete response rate was only 18%, and the median event-free survival was 9 months. Another phase II clinical trial utilized MTX induction, followed by ARA C and then chemo-mobilization, followed by high-dose therapy with Carmustine/Thiotepa and then autologous transplant [35]. The overall survival at 5 years was 69% for all patients and 87% for those that were able to have a bone marrow transplant (BMT).

A more recent study, CALGB 50202, used induction chemotherapy with MT-R: methotrexate (MTX), temozolomide, rituximab with intravenous HD-MTX (8 gm/m^2^) administered every 2 weeks × 8 and rituximab administered weekly × 6. Temozolomide (150-200 mg/m^2^) was administered starting on day + 7 and continued for 5 days and was repeated every 28 days. The patients who achieved a complete response then received intensive consolidation with cytarabine 2 gm/m^2^ BID on days 1–4 with infusional etoposide 40 mg/kg over 96 h (EA). A total of 46 patients were treated. The median progression-free survival (PFS) was 2.3 years, and the estimated PFS with 95% confidence limits at 1, 2, and 3 years were 0.64 (0.48,0.76), 0.55 (0.39,0.69), and 0.50 (0.33,0.64), respectively. The estimated overall survival (OS) with 95% confidence limits at 1, 2, and 3 years was 0.73 (0.58, 0.84), 0.71 (0.55,0.81) and 0.67 (0.51,0.79), respectively. Thus, the rates of PFS and OS in newly diagnosed PCNSL patients were at least comparable to combined modality treatment that involves reduced or standard doses of whole-brain irradiation. The MT-R-EA regimen was well-tolerated in patients age > 60 and has similar efficacy in this population as in younger patients.

In summary, it appears that high-dose MTX based therapy is beneficial in PCNSL. Stem cell transplantation may provide therapeutic benefit as well, but further studies are required.

### PVRL

Once the diagnosis of vitreoretinal lymphoma has been made, it is important to confirm that there is only ocular involvement. In the absence of systemic involvement, the question arises as to whether one eye or both eyes are involved. If there is unilateral involvement, then local therapy should be considered. It has been shown that there is no difference in recurrence rate if standard local therapy or systemic therapy is used, though there might be some difference in mortality if treatment is started earlier; but the data for that is retrospective and not definitive [12, 36, 37].

Traditionally, local therapy involved external beam radiotherapy. Up to 54 Gy has been given, but the amount that has been used has been decreased as cases of radiation retinopathy have developed [38]. Most recently, 30–36 Gy have been used; but even at these levels, radiation retinopathy can develop. Kaushik et al. have shown that at even levels of 20 Gy, radiation retinopathy can develop [39].

Since the patients now are living longer than in the 1980-1990 s, there will be more cases of subsequent radiation retinopathy. We are in the process of looking at our data, and we have found that over the last 28 years, we have treated over 70 patients that have had primary or secondary vitreoretinal lymphoma. The overall survival of patients with vitreoretinal lymphoma is now over 7 years, so whatever treatment is performed, one has to consider that these patients are living longer. Therefore, we utilize radiation primarily as salvage therapy. It should be noted, however, that others feel comfortable with the use of a median of 36 Gy [37]. The authors acknowledged that the radiated patients will all develop cataracts. In their series of 11 patients, only one eye in a patient who had diabetes developed radiation retinopathy, but another patient developed severe bilateral optic nerve atrophy. The median follow-up was 42 months, and three patients of the 11 developed recurrent intraocular disease. Several patients did not have the cataracts removed, so it is unclear if the cataract was too dense to not allow good visualization of the retina and, thus, mild retinopathy could have been missed. In addition, the treatment or outcome following the recurrent ocular disease is unclear, and no mention is made regarding the total incidence of recurrence either in the eye or brain. Weekly methotrexate (400 micrograms in 0.1 cc) injected into the vitreous cavity (intravitreal methotrexate) and was shown by Fishburne to be useful in the treatment of VRL [40]. Others have verified this finding [41]. The problem is that it takes on average 6.4 methotrexate injections to develop a remission [41]. If intravitreal methotrexate will be used, it is critical that it is the intrathecal form and that the right concentration (400 mcg/0.1 cc) be used. In addition, it is important that a paracentesis first be performed because that will decrease the chances of subconjunctival extravasation of the methotrexate. Subconjunctival extravasation causes severe irritation and pain for about 1 day. It can also result in corneal keratopathy. The aqueous paracentesis fluid can be frozen and evaluated for interleukin 10 levels to determine response to therapy [22].

In 2007, we reported the ocular toxicity studies of intravitreal rituximab [42]. We showed that 1 mg of intravitreal rituximab in 0.1 cc (the 10 mg/cc concentration) not only was safe but also could treat vitreoretinal lymphoma. This was then confirmed by others [43–45]. There are no set protocols that have been verified in larger cohorts of patients. It is important to realize as well that these patients have a high chance of local recurrence or CNS spread [12], so careful follow-up is necessary.

Traditionally, determining if there is a subtle increase in the lymphomatous cells in the vitreous between one visit and the next was the method to detect a local recurrence. Unfortunately, from 1 month to another, slight changes in the amount of vitreous cells are difficult to ascertain. We and others have shown that following the change of interleukin 10 levels in the anterior chamber is a good way to determine if the eye remains in remission [22]. Performing a paracentesis of the anterior chamber is relatively easy, and rising IL10 levels indicate the start of a local recurrence. Resuming frequent therapeutic injections (as described below) keeps the IL 10 levels in abeyance.

In the absence of large studies, for cases of monocular VRL involvement, a combination of intravitreal methotrexate and rituximab is our first line treatment. A paracentesis is performed, and the fluid is placed on dry ice and sent immediately for determination of IL10 levels. The intravitreal methotrexate is then injected, since the pressure in the eye is lowest at this point and there would be less chances of reflux. Rituximab is then injected, since a little reflux of rituximab is innocuous. During the first month, injections are performed weekly, but only the first paracentesis fluid is tested for IL10 levels. The following month, the paracentesis is again sent and, assuming that there is a decrease in IL10 levels, the injections are done every other week. In the subsequent month, levels are checked again; and if at a low level, then injections are only performed monthly. Alternatively, levels are checked and no injections are done until the IL10 levels start to rise, and then more frequent injections are resumed. The contralateral eye has to be visualized monthly to verify that it is not involved, and MRI of the brain is performed every 3 months for the first 2 years and then every 6 months, thereafter.

Again, for those patients with bilateral eye involvement, there is not an established treatment regimen. The use of bilateral radiation should be avoided because of a high probability of recurrence [12]. Systemic therapy should be considered in this situation. One regimen involves intravenous methotrexate (8 gm/m^2^ every 2 weeks initially) combined with administration of intravitreal rituximab and methotrexate. We utilize at least one series of intravitreal methotrexate and rituximab to help clear the vitreous. Again, IL10 levels in the aqueous are useful to evaluate on a monthly basis to make sure that there is no evidence of elevation. In addition, MRI of the brain every 3 months should be performed for the first 2 years.

The group that used bilateral radiation as discussed above also gave systemic therapy. We do not know the overall incidence of recurrence, but we do know that of 11 patients, 3 had ocular recurrence during a median of 42 months of follow-up. Their treatment protocol was treatment with R-MPV (rituximab 375 mg/m^2^ intravenously (IV) on day one, methotrexate 3.5 g/m^2^ IV over 2 h on day one followed by leucovorin rescue, procarbazine 100 mg/m^2^/day orally for 7 days with odd cycles only, and vincristine 1.4 mg/m^2^ capped at 2 mg IV on day one) given every 2 weeks for five cycles. RT was started a minimum of 2 weeks after the last cycle of methotrexate and targeted the bilateral orbits, including the entire globes. At least 1 month after the completion of RT, two cycles of rituximab (375 mg/m^2^ IV on day one) and cytarabine (3 g/m^2^ IV over 3 h once daily on days one and two) were administered on two occasions, 2 weeks apart. Intrathecal (IT) methotrexate (12 mg intrathecal or 6 mg by Ommaya reservoir) or cytarabine (100 mg intrathecal) was typically administered once per cycle. We do not feel that Omaya reservoirs are needed or should be used.

### Emerging therapies

#### Stem cell transplantation

There exists limited data on the use of high-dose chemotherapy and autologous stem cell transplantation in the treatment of PVRL, with most trials either including only PCNSL or combining PCNSL with PVRL. The following two studies included only patients with PCNSL: In 2003, a publication from Memorial Sloan Kettering described 28 patients with PCNSL who underwent intravenous methotrexate and Ara-C followed by BEAM conditioning chemotherapy and autologous stem cell transplantation [12]. The median follow-up was 9.3 months. There was an 18% complete response rate with a median event-free survival of 9 months. A subsequent study from Germany enrolled 30 patients who were younger than 65 years of age who had had PCNSL. Patients underwent high-dose methotrexate induction and ARA-C, then stem cell mobilization, followed by Carmustine and Thiotepa high-dose therapy and autologous stem cell transplantation, and then whole brain radiotherapy [35]. Of those that were able to successfully undergo the transplant, there was an 87% overall survival rate at 5 years. When examining all enrolled patients, including those that could not complete the high-dose methotrexate, there was a 69% survival rate at 5 years. Other different induction and conditioning regimens in the treatment of PCNSL have been described as well [35, 46, 47].

The use of stem cell transplantation for PVRL has been described [48]. Originally, it was used for refractory or recurrent cases of vitreoretinal and/or CNS lymphoma. The patients that were able to complete the treatment regimen had a median overall survival was 58.6 months compared to 18.3 months for the overall group. The two-year survival rate was 69% for the transplant group compared to 45% for the entire group [48]. Other conditioning chemotherapy regimens have been used, but it appears that this might be a reasonable way to treat refractory or recurrent PVRL [49, 50].

At the present time, we believe that this is a very reasonable treatment for patients with refractory or recurrent PVRL or PCNSL. Originally, it was used for patients 65 years or younger; the age of the patient is not as important as the systemic health of the patient [48]. Whether high-dose chemotherapy and autologous stem cell transplantation should be incorporated as consolidation in primary treatment of PVRL remains debatable, but it is not unreasonable to consider.

#### Pomalidomide

The use of thalidomide-related agents in the treatment of systemic diffuse large B cell lymphoma has been described [51, 52]. Pomalidomide (a similar agent but with better penetration into the CNS) has shown efficacy in an animal model of CNS lymphoma [53]. An ongoing study being conducted at Mayo Clinic sites and Dana-Farber Cancer Institute was developed to investigate the use of pomalidomide for refractory or recurrent CNS or vitreoretinal. Enrollment continues with promising results [54].

#### Ibrutinib

The fact that the MYD88 L265P mutation is so prevalent in cases of vitreoretinal lymphoma has opened a new possible treatment option. Ibrutinib was originally utilized for the treatment of chronic lymphocytic leukemia (CLL) [55]. It is an oral targeted agent whose mechanism of action is inhibiting Bruton’s tyrosine kinase (BTK). In addition, it inhibits the HCK tyrosine kinase protein. Interestingly, both of these pathways are upregulated by the MYD88 L265P mutation [56]. Ibrutinib is approved for the treatment of Waldenström’s macroglobulinemia WM [57]. Over 90 + % of WM patients have the MYD88 mutation [58]. Recently, it has been shown that ibrutinib has an 80% response rate for the systemic (ABC) activated B cell form of DLBCL [59].

An interim analysis of a phase II clinical trial utilizing single agent ibrutinib in relapsed or refractory PCNSL and PVRL was recently presented [60]. Eleven patients with PCNSL and seven patients with PVRL at time of study entry were included in the analysis. The median number of treatment cycles was five, with an overall response rate of 56% and 3 of 18 patients achieving a CR as best response. The FDA has approved ibrutinib for Waldenström’s macroglobulinemia. In two patients with WM who subsequently developed VRL followed at the Mayo Clinic, Rochester, MN, we have treated with ibrutinib in a salvage setting with complete remissions in both, however, one developed resistance to the ibrutinib and developed recurrence for which he was resumed on local intravitreal infections (unpublished data). Because of the fact that Waldenström’s and primary vitreoretinal lymphoma both have a similar MYD88 mutation, it might be reasonable to use this agent in clinical trials for patients with VRL. The fact that it is orally delivered and that it can penetrate the blood retinal and blood CNS barriers makes it an attractive agent that requires study for vitreoretinal lymphoma [61].

Caution should be taken when incorporating ibrutinib with high-dose steroids, however, as cases of invasive fungal and pneumocystis infections have been reported in this setting [62, 63].

#### Lenalidomide plus rituximab

A recent abstract presented at the American Society of Hematology Annual Meeting in 2016 from the French LOC Network reviewed results from a phase II trial of lenalidomide plus rituximab for relapsed or refractory PCNSL or PVRL [60]. Of the 45 enrolled patients, 9 patients had PVRL and 10 patients had secondary VRL; 6 of the 19 patients achieved a complete remission. Thus, immunomodulatory agents appear to be very promising in the treatment of VRL.

#### Vitrectomy

As mentioned previously, it may be that debulking could have a therapeutic effect as well, though there are only a few case reports. At the present time, there is not enough data to consider this as the only therapy, but it might be a useful adjunct in cases with significant cellular load [14, 15].

### Summary of therapy by authors

**Fig. 3:**
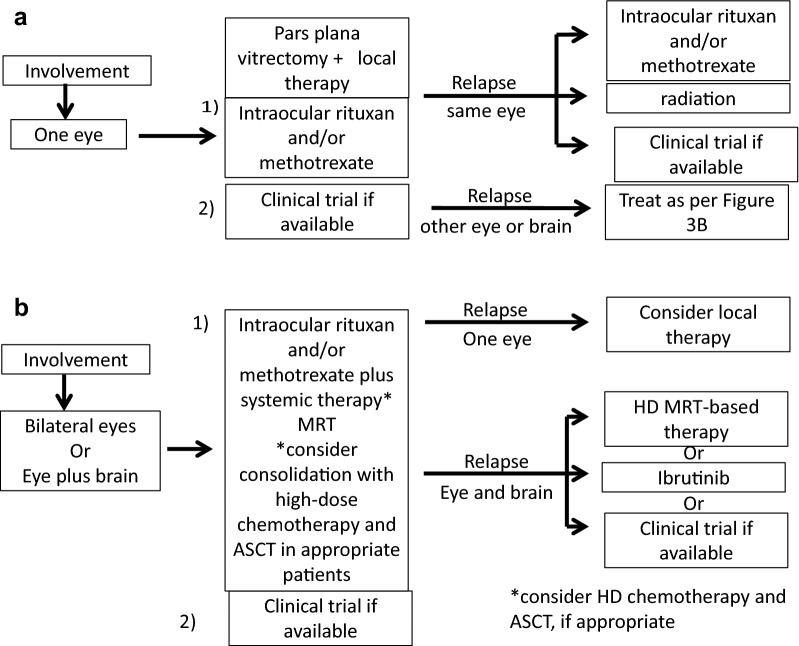
Algorithm for treatment strategies for vitreoretinal lymphoma at Mayo Clinic. *MRT* methotrexate, rituximab, temozolomide, *HD MRT* high-dose methotrexate, rituximab, temozolomide, *ASCT* autologous stem cell transplant

Figure 3 summarizes our suggestions for vitreoretinal lymphoma. In the presence of PVRL and no evidence of CNS lymphoma, if it is unilateral, then consideration of local intravitreal therapy should be given. If local recurrence occurs again in the absence of bilateral involvement or CNS involvement, either resumption of local intravitreal therapy or local radiation (30 Gy) can be given. If there is bilateral vitreoretinal recurrence and/or CNS involvement, treatment with ASCT can be given in conjunction with local therapy. If the patient is not systemically capable of undergoing stem cell transplantation, systemic therapy with ibrutinib can be considered if there is evidence of MYD88 L265P mutation.

If there is initial bilateral ocular involvement with PVRL then systemic methotrexate, rituximab and temozolomide could be used in conjunction with local intravitreal therapy with rituximab and methotrexate. If there is bilateral recurrence of CNS involvement, ASCT can be considered. If initially there is VRL with CNS involvement, then systemic therapy with methotrexate and rituximab should be given. Local therapy with intravitreal methotrexate and rituximab can be given for the first month as well. If recurrence occurs, transplantation can be done if the patient is less than 70 and is appropriate for high-dose chemotherapy and stem cell transplantation. If the patient is older or in poor health and if the cells have the MYD88 L265P mutation, besides local therapy, systemic ibrutinib can be considered.

## Conclusions

Many advances in the detection and diagnosis of PVRL have been made in the recent past with new technologies such as NGS developing in our diagnostic armamentarium. Although historically, very limited treatment options existed for PVRL, new therapeutic options, including intravitreal therapy, systemic chemotherapy, and immunomodulatory agents are changing the landscape of treatment options for this rare disease.
