# Correction to: The diagnosis and treatment of primary vitreoretinal lymphoma: a review

**DOI:** 10.1186/s40942-018-0126-y

**Published:** 2018-06-04

**Authors:** Jose S. Pulido, Patrick B. Johnston, Grzegorz S. Nowakowski, Alessia Castellino, Harish Raja

**Affiliations:** 1 Department of Ophthalmology, Mayo Clinic, 200 First Street, SW, Rochester, MN 55905 USA; 2 Departments of Hematology, Mayo Clinic, Rochester, MN USA; 3 Department of Molecular Medicine, Mayo Clinic, 200 First Street, SW, Rochester, MN 55905 USA

## Correction to: Int J Retin Vitr (2018) 4:18 10.1186/s40942-018-0120-4

Following publication of the original article [1], the authors reported that one of the authors’ name is spelled incorrectly. In this correction, the incorrect and correct author names are shown.

Originally, the author name was published as:

- Allessia Castellino.

The correct author name is:

- Alessia Castellino.
